# S100 and CD34 positive spindle cell tumors of the uterine cervix with *EGFR* mutation: a hitherto unrecognized neoplasm phenotypically and epigenetically overlapping with “*NTRK*-rearranged spindle cell neoplasms” of the uterus

**DOI:** 10.1007/s00428-024-03936-z

**Published:** 2024-10-10

**Authors:** Michael Michal, Josef Kuruc, Veronika Hájková, Květoslava Michalová, Natálie Klubíčková

**Affiliations:** 1 Bioptical Laboratory, Ltd., Pilsen, Czech Republic; 2 Department of Pathology, Medical Faculty and Charles University Hospital Plzen, Charles University, Alej Svobody 80, 323 00 Pilsen, Czech Republic; 3 Cytopathos, Ltd., Bratislava, Slovakia

**Keywords:** Uterine cervix, Soft tissues, NTRK-rearranged spindle cell neoplasms, Perivascular and stromal hyalinization, S100 and CD34 co-expression, EGFR mutation

## Abstract

*NTRK*-rearranged spindle cell neoplasm represents an emerging entity included in the latest 5th edition of WHO classification of both soft tissue and female genital tumors. By immunohistochemistry, they are commonly positive for CD34, S100 protein, and CD30 and typically harbor fusions of kinase genes such as *NTRK1/2/3*, *RET*, and *BRAF*. In the gynecological tract, they typically affect the uterine cervix or uterine body. Most of the reported cases had fibrosarcoma-like morphology, occasionally showing perivascular and stromal hyalinization with only a few cases showing a less cellular spindle cell proliferation. Except for one case with *RET* fusion, all other gynecological cases harbored exclusively *NTRK1/2/3* fusions. Besides kinase gene fusions, the analogous tumors in soft tissues may also harbor activating *EGFR* or *BRAF* point mutations, but no such case has been described in the uterus. Herein we are reporting two cases from the uterine cervix showing morphology and molecular features previously unreported at this anatomic site. The patients were 46 and 34 years old and clinically presented with unremarkable cervical polyps each measuring 8 mm in diameter. Histologically, both cases had a rounded polypoid outline and were composed of hypocellular proliferation of bland spindle cells lacking mitotic activity and growing in a fibrotic stroma which was punctuated by prominent small vessels with thick hyalinized walls. Immunohistochemically, both showed a diffuse expression of CD34, CD30, and S100 protein, whereas SOX10 was negative. Both cases harbored exon 20 *EGFR* mutation and did not reveal any fusions or significant copy number changes. The patient in case 1 was treated by hysterectomy with salpingectomy with no other residual tumor detected, and she was alive and well 27 months after the diagnosis. The patient in case 2 had no other known tumors at the time of diagnosis, but no follow-up is available. We believe the reported cases represent a hitherto unrecognized variant of “*NTRK*-rearranged spindle cell neoplasms” of the uterine cervix with novel *EGFR* mutations.

## Introduction

Fusions of *NTRK1/2/3*, *RET*, *BRAF*, *RAF1*, *ALK*, and other kinase genes involved in the RAS-RAF-MAPK signaling cascade have been increasingly recognized in the group of spindle cell soft tissue tumors with largely overlapping morphology and immunophenotype [1]. The tumors often consist of proliferation of monomorphic spindle cell with perivascular and/or stromal hyalinization which, by immunohistochemistry, are commonly positive for CD34, S100 protein, and CD30 [1–7]. Instead of gene fusions, a small subset of these tumors in soft tissues or kidneys (in the latter site a traditional name congenital mesoblastic nephroma is often used) may harbor activating *EGFR* or *BRAF* point mutations [8–12]. As has been relatively recently reported, morphologically and immunophenotypically identical tumors with *NTRK1/2/3* or *RET* fusions may also occur in the uterus and the 5th edition of WHO classification of both bone and soft tissue tumors as well as female genital tumors coined them *NTRK*-rearranged spindle cell neoplasms [13–18]. However, molecular aberrations other than gene fusions associated with these tumors in soft tissue sites have so far not been reported in the gynecological tract. Herein we are reporting two unusual cases from the uterine cervix which show morphology and molecular features previously unreported at this anatomic site, and which further underscore the overlapping morphological, immunohistochemical, and molecular genetic features shared between this group of tumors in soft tissues and in the uterus.

## Materials and methods

### Case selection

Both cases were identified in the author’s files based on overlapping clinicopathologic and molecular features. One of them (case 1) was a consultation, whereas the other was encountered during a routine histopathological practice. Case 1 was published previously as part of a larger series on tumors with kinase gene alterations [5]. Clinical features were abstracted from the medical records and follow-up obtained from the attending physicians.

To identify more cases as well as to assess the overall incidence of this neoplasm, 499 consecutive routine histopathological cases accessed at Biopticka Laboratory Ltd. from 12/2023–3/2024 (the period directly predating the identification of case 2) and diagnosed as cervical polyp (identified by searching for cases with the ICD-10 diagnosis N84.1 in our LIS) were morphologically screened for overlapping microscopic features. Most of these cases consisted of common endocervical polyps or fibroepithelial stromal polyps and less commonly represented other benign polypoid spindle cell lesions. In the next step, cases with the most similar morphology (*n* = 34) were stained with CD34, CD30, and S100 immunohistochemistry to confirm or exclude the diagnosis. Additionally, all cases labeled with keywords “cervix + polyp” (*n* = 51) in our consultation tumor registry were also reviewed to identify further cases.

### Immunohistochemistry

Immunohistochemistry for CD34 (QBEnd/10, Dako, 1:200), S100 protein (polyclonal, Ventana, premixed), CD30 (Ber-H2, Dako, premixed), and SOX10 (SP276, Ventana, 1:100) was performed using a Ventana BenchMark ULTRA (Ventana Medical System, Inc., Tucson, Arizona). Other immunohistochemical markers ordered during the initial work-up of case 1 included HMB45 (HMB45, Dako, 1:400), CK AE1-3 (AE1/AE3, Dako, premixed), SMA (1A4, Dako, 1:500), and EMA (E29, Dako, 1:400).

### Molecular genetic studies

For molecular genetic testing, case 1 was analyzed using the commercially available CTL FusionPlex kit (ArcherDX Inc., Boulder, CO) which detects fusion transcripts in selected exons of 18 genes (including *ALK*, *BRAF*, *NTRK1/2/3*, and *RET*) and mutations in hotspots of another 18 genes [3]. Additionally, methylation analysis was performed on this case as well. Briefly, 250 ng of genomic DNA was extracted from the paraffin block and was subjected to bisulfite conversion and processed on the Illumina Infinium Methylation EPIC/850 k platform with over 850,000 methylation sites according to the manufacturer’s instructions [5]. The idat raw data files were uploaded to a DNA methylation-based classification tool DKFZ Sarcoma classifier v10.1 available via https://www.molecularneuropathology.org/ [19, 20]. Case 2 was analyzed with the commercially available TruSight Oncology 500 panel (Illumina, San Diego, CA, USA), a comprehensive DNA and RNA NGS assay that identifies somatic variants, copy number changes, tumor mutational burden, and fusions [5]. The original set of probes for fusion detection (RNA part of the assay) was replaced with the commercially available TruSight RNA Pan-Cancer Panel (Illumina) targeting 1385 genes to achieve a broader coverage.

## Results

### Clinicopathological features of the study cohort

**Fig. 1:**
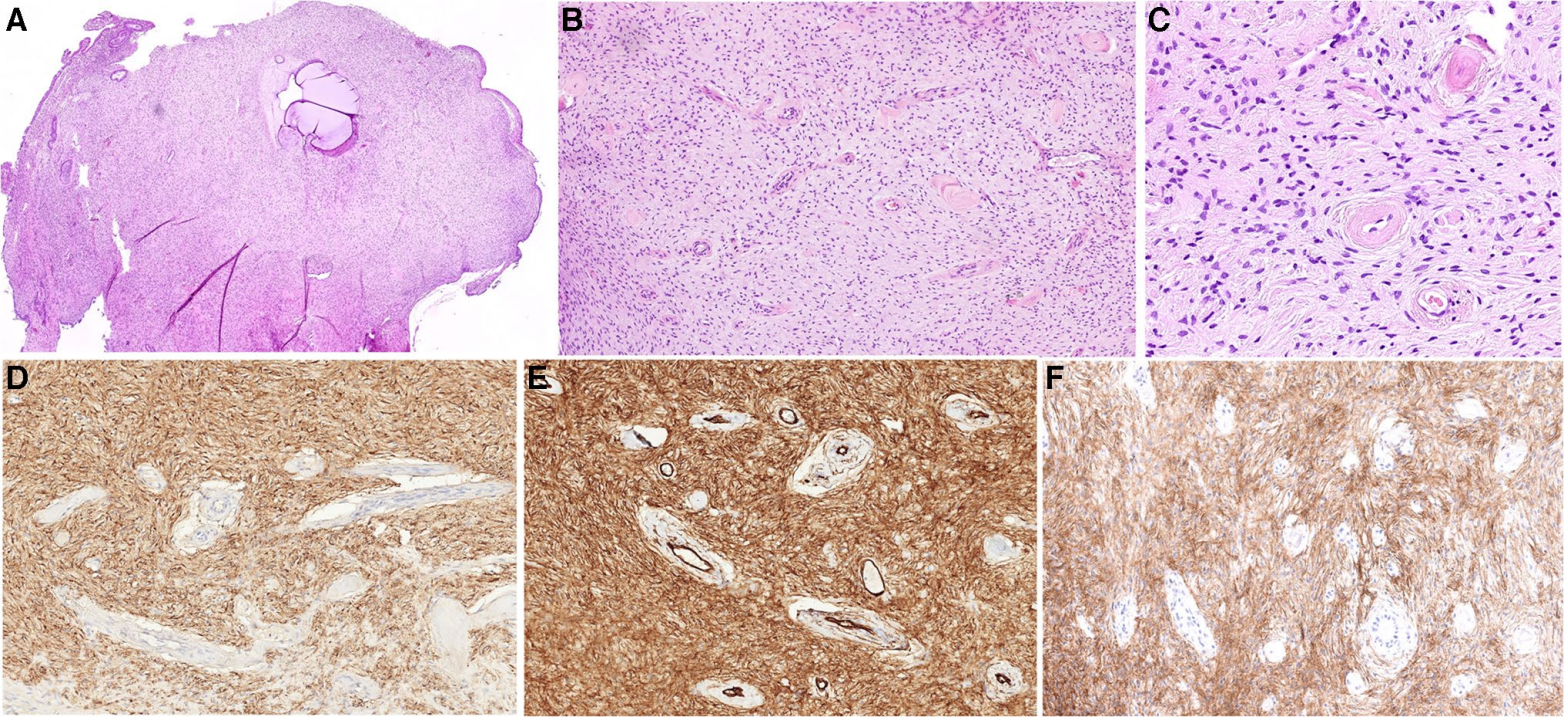
Case 1 had a rounded polypoid outline partially covered by non-dysplastic endocervical mucosa. Variably dilated benign endocervical glands were occasionally present, including a small Nabothian cyst in the center (**A**). The tumor consisted of a hypocellular proliferation of bland spindle cells lacking any mitotic activity and growing in a fibrotic stroma punctuated by prominent small vessels with thick hyalinized walls (**B**,** C**). Immunohistochemical staining with S100 protein (**D**), CD34 (**E**), and CD30 (**F**) revealed diffuse strong expression

**Fig. 2:**
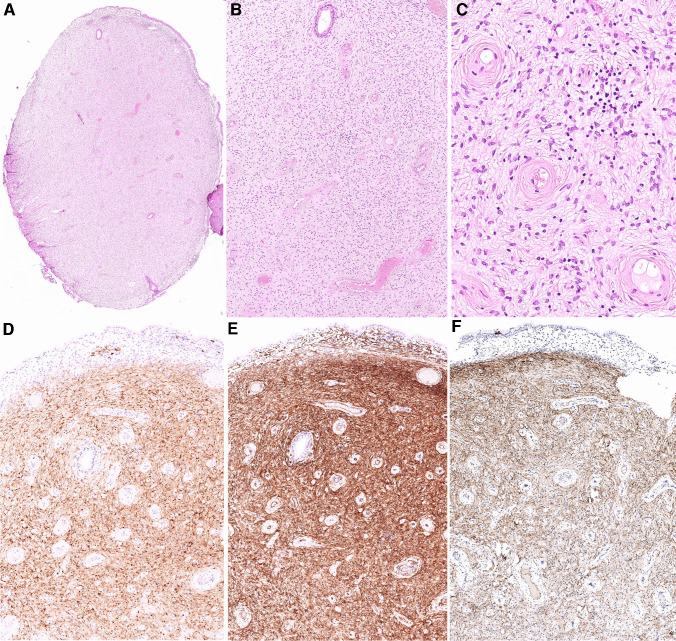
Case 2 had a similar rounded polypoid outline, and its surface was partially covered by non-dysplastic endocervical mucosa (**A**). The tumor was again composed of hypocellular proliferation of bland spindle cells lacking any mitotic activity and growing in a fibrotic stroma punctuated by prominent small vessels with thick hyalinized walls. Variably dilated benign endocervical glands were occasionally present as well (**B, C**). Immunohistochemically, it showed a diffuse expression of S100 protein (**D**), CD34 (**E**), and CD30 (**F**)

The clinicopathological and molecular features of the two cases are summarized in Table 1. Both tumors clinically presented as unremarkable small polypoid cervical lesions during a regular gynecological screening examination, and both were incompletely excised. The patients were 46 and 34 years old, and both tumors measured 8 mm in diameter. Histologically, both cases appeared nearly identical. They had a rounded polypoid outline and were composed of hypocellular proliferation of bland spindle cells growing in a fibrotic stroma punctuated by prominent small vessels with thick hyalinized walls. Variably dilated benign endocervical glands were occasionally present as well. No mitotic activity was detected. The surface of the lesions was partially covered by non-dysplastic endocervical mucosa (Figs. 1A–C and 2A–C). Immunohistochemically, both cases showed a diffuse expression of S100 protein, CD34, and CD30 (Figs. 1D–E and 2D–E), whereas SOX10 was negative. Other immunohistochemical markers tested in case 1, namely HMB45, CK AE1-3, SMA, and EMA, were negative.

**Table 1:** Clinicopathological features of the 2 studied cases

	Age (years)	Size	Clinical presentation	IHC +	IHC-	Molecular features	Follow-up
Case 1	46	8 mm	Cervical polyp	S100, CD34, CD30	SOX10, HMB45, CK AE1-3, SMA, EMA	*EGFR* [c.2303_2311dup, p.(S768_D770dup)], (AF: 24%); *BRAF* [c.1798_1799delinsAA, p.(Val600Lys)] (AF: 9%); no fusions or CNV	NED (27 months)
Case 2	34	8 mm	Cervical polyp	S100, CD34, CD30	SOX10	*EGFR* [c.2310_2311insGGT, p.(D770_N771insG)], (AF: 28%); *ATM* [c.3850del, p. (T1284Q*X9)], (AF: 49%); no fusions or CNV	NA

*IHC* Immunohistochemistry, *SMA* Smooth muscle actin, *EMA* Epithelial membrane antigen, *AF* Allelic frequency, *CNV* Copy number variation, *NED* No evidence of disease, *NA* Not available

The patient in case 1 underwent hysterectomy with salpingectomy with no other residual tumor detected. She was alive and well 27 months after the diagnosis. At the time of diagnosis, the patient in case 2 had no other known tumors, but the case is too recent for any meaningful follow-up.

### Molecular genetic features of the study cohort

**Fig. 3:**
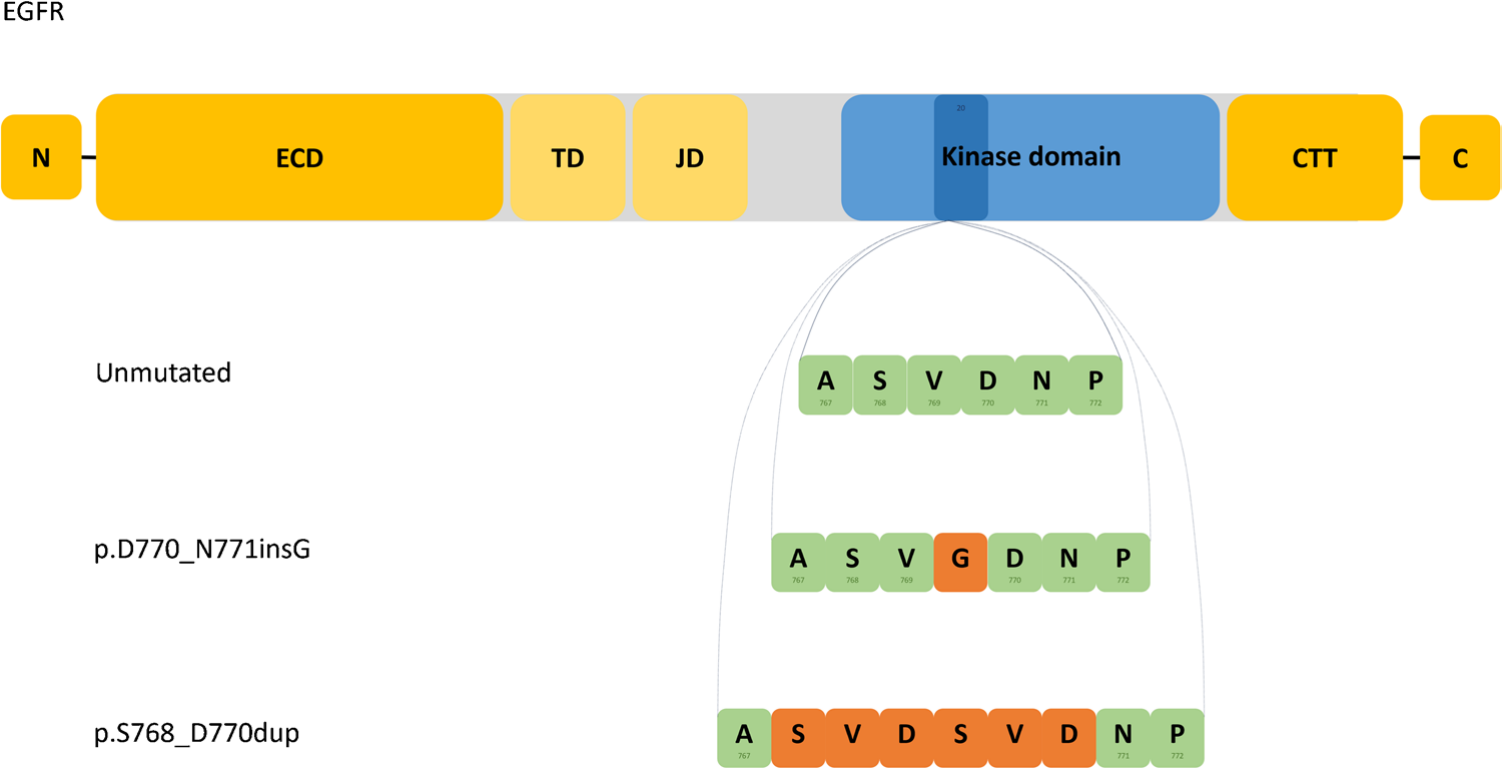
The amino-acyl sequence of the p.676–772 region in exon 20 of the unmutated *EGFR* kinase domain is depicted in the top green row. The *EGFR* p.S768_D770dup present in case 1 is illustrated at the bottom, whereas the *EGFR* p.D770_N771insG detected in case 2 is shown in the middle. Abbreviations: CTT, C-terminal tail; dup, duplication; ECD, extracellular domains; ins, insertion; JD, juxtamembrane region; TD, transmembrane region. One-letter code for amino-acyl groups in the kinase domain is used

**Fig. 4:**
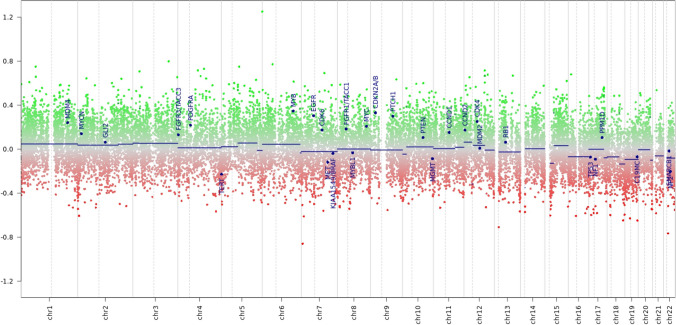
Copy number variation plot obtained from the DNA methylation data in case 1 did not disclose any significant copy number changes

The molecular testing in case 1 revealed a concurrent *EGFR* [c.2303_2311dup, p.(S768_D770dup)] mutation (Fig. 3) with an allelic frequency of 24% and *BRAF* [c.1798_1799delinsAA, p.(Val600Lys)] mutation with an allelic frequency of 9%. Case 2 harbored an *EGFR* [c.2310_2311insGGT, p.(D770_N771insG)] mutation (Fig. 3) with an allelic frequency of 28% and *ATM* [c.3850del, p. (T1284Q*X9)] mutation with allelic frequency of 49%, therefore most likely of germline origin. No fusions were detected in none of the cases. The methylation profile in case 1 did not match with a high calibrated score to any of the methylation classes in the DKFZ sarcoma methylation classifier [20]. Additionally, the copy number variation plot obtained from the DNA methylation data did not disclose any significant copy number changes (Fig. 4). Case 2 showed a low tumor mutational burden (3,9 mut/Mb) and no significant copy number changes in the analyzed genes.

### Review of other cervical polyps

**Fig. 5:**
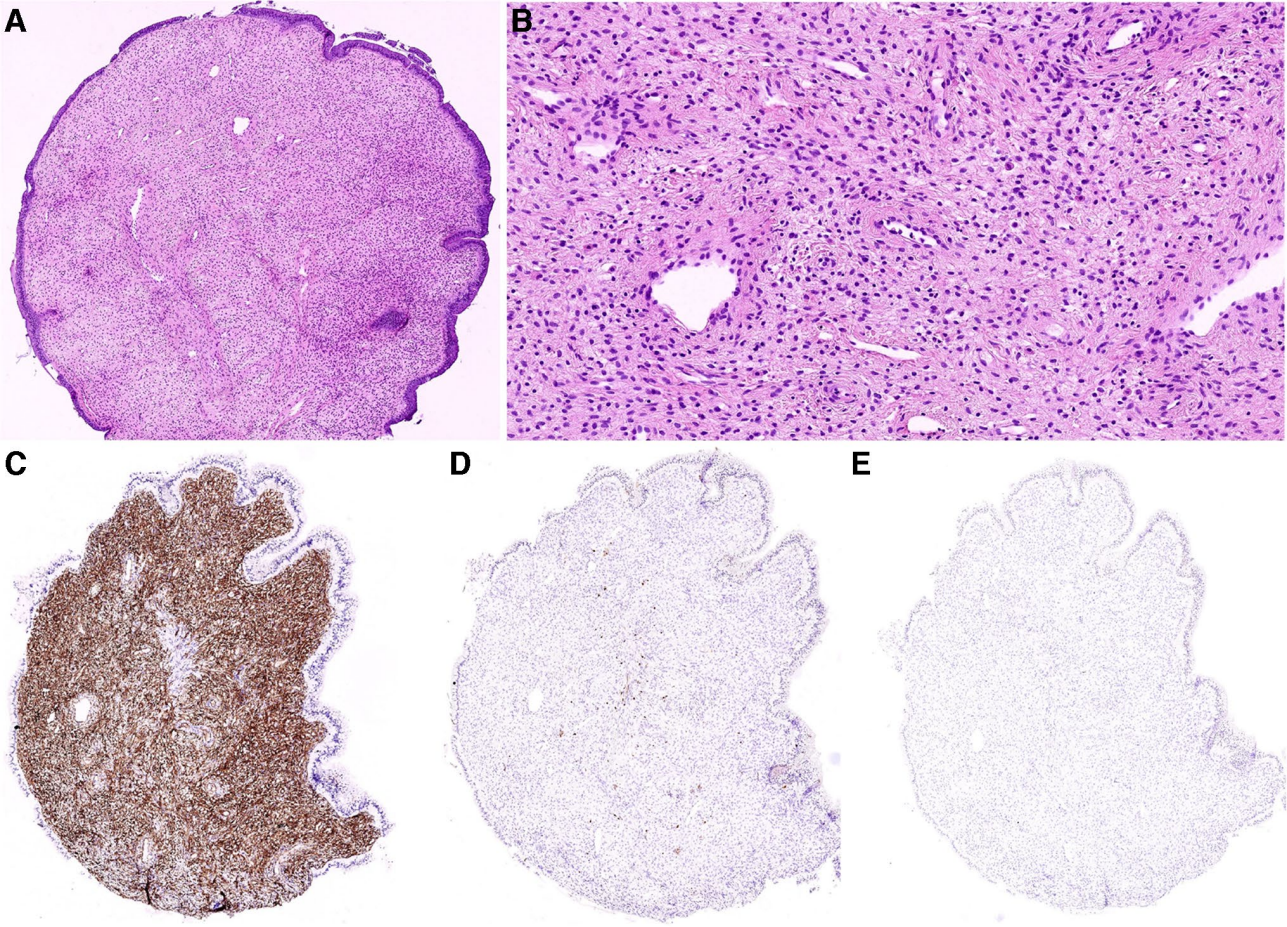
None of the other 499 consecutive routine surgical pathology cases which were reviewed had morphological features identical to the studied tumors with *EGFR* mutations. Whereas some cases had a similar rounded outline and were composed of bland spindled cells growing within a sclerotic stroma such as the depicted fibroepithelial stromal polyp, none of them exhibited the prominent hyalinized vasculature changes (**A**, **B**). While all tested cases including one of the fibroepithelial stromal polyps (shown here) were positive for CD34 (**C**), none of the 34 cases with the most similar morphological features which were subsequently analyzed by immunohistochemistry showed expression of S100 protein (**D**) and CD30 (**E**)

None of the other 499 consecutive routine surgical pathology cases diagnosed as cervical polyps at Biopticka Laboratory Ltd. which were reviewed had morphological features identical to the studied tumors with *EGFR* mutations. Whereas some cases had a similar rounded outline and were composed of bland spindled cells growing within a sclerotic stroma, none of them exhibited the prominent hyalinized vasculature changes (Fig. 5A, B). While all tested cases were positive for CD34 (Fig. 5C), none of the 34 cases with the most similar morphological features which were subsequently analyzed by immunohistochemistry showed expression of S100 protein and CD30 (Fig. 5D, E). As a result, the incidence of the studied lesions was lower than 1 in 500 (< 0.2% of cervical polyps) based on our morphological analysis.

Similarly, none of the 51 cases from our tumor registry coded as “cervix + polyp” showed a morphology consistent with the studied case.

## Discussion

*NTRK*-rearranged spindle cell neoplasm represents an emerging entity included in the latest 5th edition of WHO classification of female genital tumors. These mesenchymal tumors predominantly affect the uterine cervix with only about 15% of cases occurring in the uterine corpus. They only rarely present as a cervical polyp [17, 18], and their average size is 6.9 cm (range, 1.3–23 cm) [17]. Most of the cases reported in the uterus had fibrosarcoma-like morphology, occasionally showing perivascular and stromal hyalinization with only a few cases showing a less cellular spindle cell proliferation [16–18, 21]. However, we are not aware of any published case with such a small size and hypocellular, bland histology lacking any mitotic activity as was encountered in the herein presented cases. It is therefore possible these benign clinicopathological features in the uterus are more commonly or always associated with the *EGFR* mutations detected in both our cases since to our knowledge, except for one case with *RET::SPECC1L* fusion [14], all other reported cases harbored exclusively *NTRK1/2/3* fusions [17]. Our cases are thus the first two published cases falling within the spectrum of *NTRK*-rearranged spindle cell neoplasms of the gynecological tract which instead of fusions are driven by activating mutations of the kinase genes. On the other hand, tumors with similar bland hypocellular morphology and various kinase gene fusions have been described in soft tissues [1–3, 5, 6]. Besides the characteristic morphological and immunohistochemical features, the notion that the herein presented cases indeed represent a variant of *NTRK*-rearranged spindle cell neoplasms is further reinforced by our recently published methylation analysis which included case 1 as well as 8 other kinase-fusion-associated soft tissue and uterine tumors. Unsupervised clustering by T-distributed stochastic neighborhood embedding as well as unsupervised hierarchical clustering of their methylation profiles showed that case 1 clustered together with most other analyzed tumors with fusions of *NTRK1/3*, *BRAF*, *RET*, and others [5], including one previously published uterine case with *STRN::NTRK3* fusion [16].

The *EGFR* mutations detected in our cases occurred in exon 20 of *EGFR*. Both the small-scale duplication and one-nucleotide insertion in cases 1 and 2, respectively, involved the αC helix and the αC-β4 loop in the tyrosine-kinase domain of the protein, likely rendering the kinase domain constitutively active [22]. Internal tandem duplications of the whole *EGFR* kinase domain have been repeatedly reported in some pediatric fusion-negative spindle cell neoplasms morphologically consistent with infantile fibrosarcoma/congenital mesoblastic nephroma [9, 11, 12]. Nevertheless, these aberrations involved the whole kinase domain spanning exons 18–25. In contrast, while small-scale exon 20 mutations of *EGFR* as detected in our 2 cases are common in epithelial tumors, e.g., non-small lung carcinoma [23], until recently, such alterations in mesenchymal tumors have been reported only in fibrous hamartoma of infancy [24]. However, a very recent paper by Vallese et al. has also described similar alterations in three mesenchymal tumors with a variable bland spindle cell morphology and S100 protein (2/3 cases) and CD34 (3/3 cases) co-expression. While the morphology of these tumors was not identical to the herein presented cervical cases, they also seem to fit well within the expanding spectrum of *NTRK*-rearranged spindle cell neoplasms [10].

In addition to *EGFR* mutation, case 1 also harbored a *BRAF* V600K mutation which affects the kinase domain of *BRAF* and increases its activity. Similar mutations are found in a myriad of tumors, including some soft tissue tumors [25]. Notably, *BRAF* fusions and point mutations including p.V600E/D were also detected in cases of infantile fibrosarcoma [8]. Given the presence of recurrent aberrations of *EGFR* in both our cases and the very low allelic frequency of the *BRAF* mutation, the latter probably represents just an additional genetic event in a lesion primarily driven by the *EGFR* mutation. In case 2, a frameshift *ATM* mutation was detected and caused the truncation and destabilization of ATM protein. Since ATM is a protein kinase essential for double-strand DNA break repair [26], the mutation may have rendered the affected cells more prone to serious DNA damage, eventually enabling the emergence of other mutations. However, we do not think it was the key oncogenic driver per se as it was likely of germline origin and was detected in only one of the two presented cases which both harbored functionally similar *EGFR* exon 20 mutations. Moreover, the latter are known to be strong driving pathogenic alterations in multiple neoplasms including those with clinicopathological features overlapping with the presented cases as discussed above.

The finding of activation point mutations in this group of uterine mesenchymal tumors has two implications. The first is related to the choice of gene panel used for molecular testing of tumors from the morphological spectrum of *NTRK*-rearranged neoplasms which should now also cover gene mutations to avoid false negative result. The second is related to therapy. A significant proportion of *NTRK*-rearranged spindle cell neoplasms of the uterus have followed an aggressive clinical course [13, 15, 17]. While the herein presented cases are most likely benign, the overall number of clinically tested and, accordingly, published cases of uterine *NTRK*-rearranged spindle cell neoplasms is low, and it is possible that a malignant variant with *EGFR* mutation simply awaits its first publication. Patients with such cases might then benefit from targeted treatment with EGFR small molecule inhibitors.

The differential diagnosis includes other polypoid cervical lesions composed of bland spindle cells, primarily ordinary cervical polyps, fibroepithelial stromal polyps, and superficial myofibroblastomas. While all these lesions may be composed of similar benign-appearing spindled cells with abundant small vessels, we found the most useful distinguishing morphological feature to be the presence of hyalinized vessels which was found exclusively in the herein presented 2 cases. The studied tumors seem to be exceedingly rare, representing less than 0.2% of cervical polyps based on our morphological review of 500 consecutive cases diagnosed as endocervical polyp. When needed, immunohistochemistry for S100 protein and/or CD30 can be used to confirm or exclude the diagnosis. In contrast, CD34 is not discriminatory, as it is typically expressed in all 3 lesions included in the differential diagnosis. Lastly, a recent study introduced a novel group of uterine sarcomas with *ERBB2/ERBB3* alterations which also show diffuse S100 positivity and may present as a cervical polyp. However, in contrast to the herein presented cases, these tumors are morphologically high-grade sarcomas and are also consistently SOX10 positive [27].

In summary, we report two cases of S100 and CD34 positive polypoid spindle cell tumors of the uterine cervix harboring *EGFR* mutation which we believe represents a hitherto unrecognized variant of “*NTRK*-rearranged spindle cell neoplasms” of the uterus. Our cases expand the morphological and molecular spectrum of this emerging WHO entity of the lower gynecological tract.

## Data Availability

The complete datasets generated during the current study are available from the corresponding author upon reasonable request.
